# Burden of *Staphylococcus aureus* infections after orthopedic surgery in Germany

**DOI:** 10.1186/s12879-020-04953-4

**Published:** 2020-03-19

**Authors:** Fraence Hardtstock, Kirstin Heinrich, Thomas Wilke, Sabrina Mueller, Holly Yu

**Affiliations:** 1 Ingress-Health, Alter Holzhafen 19, 23966 Wismar, Germany; 2 Pfizer, Inc., Collegeville, PA USA; 3 IPAM, University of Wismar, Wismar, Germany

**Keywords:** *Staphylococcus aureus*, Postoperative surgical site infections, Healthcare resource utilization, Claims data

## Abstract

**Background:**

This study assessed incidence, risk factors, and outcomes of *Staphylococcus aureus* infections (SAI) following endoprosthetic hip or knee, or spine surgeries.

**Methods:**

Adult patients with at least one of the selected surgeries from 2012 to 2015 captured in a German sickness fund database were included. SAI were identified using *S. aureus*-specific ICD-10 codes. Patients with certain prior surgeries and infections were excluded. Cumulative incidence and incidence density of post-surgical SAI were assessed. Risk factors, mortality, healthcare resource utilization and direct costs were compared between SAI and non-SAI groups using multivariable analyses over the 1 year follow-up.

**Results:**

Overall, 74,327 patients who underwent a knee (28.6%), hip (39.6%), or spine surgery (31.8%) were included. The majority were female (61.58%), with a mean age of 69.59 years and a mean Charlson Comorbidity Index (CCI) of 2.3. Overall, 1.92% of observed patients (20.20 SAI per 1000 person-years (PY)) experienced a SAI within 1 year of index hospitalization. Knee surgeries were associated with lower SAI risk compared with hip surgeries (Hazard Ratio (HR) = 0.8; *p* = 0.024), whereas spine surgeries did not differ significantly from hip surgeries. Compared with non-SAI group, the SAI group had on average 4.4 times the number of hospitalizations (3.1 vs. 0.7) and 7.7 times the number of hospital days (53.5 vs. 6.9) excluding the index hospitalization (*p* < 0.001). One year post-orthopedic mortality was 22.38% in the SAI and 5.31% in the non-SAI group (*p* < 0.001). The total medical costs were significantly higher in the SAI group compared to non-SAI group (42,834€ vs. 13,781€; *p* < 0.001). Adjusting for confounders, the SAI group had nearly 2 times the all-cause direct healthcare costs (exp(b) = 1.9; *p* < 0.001); and 1.72 times higher risk of death (HR = 1.72; *p* < 0.001).

**Conclusions:**

SAI risk after orthopedic surgeries persists and is associated with significant economic burden and risk of mortality. Hence, risk reduction and prevention methods are of utmost importance.

## Background

Postoperative surgical site infections (SSIs) are associated with increased morbidity and mortality, decreased quality of life for patients [1–4], and higher hospitalization costs compared with surgery patients without such infections [4–6]. Previous studies have found 20% of SSIs are *Staphylococcus aureus* (*S. aureus*), making it the most common SSI pathogen identified [7–9]. As the number of orthopedic surgeries annually increases [10, 11], negative outcomes for post-surgical infections are of great concern, particularly as SSIs persist despite infection control measures [12]. In Germany nearly 251 hip replacements and 180 total knee replacement surgeries per 100,000 inhabitants occur annually, representing one of the highest frequencies among EU Member States [13, 14]. One German study found an SSI rate of 0.98% following orthopedic surgeries with *S. aureus* causing approximately one-third [5]. Previous studies of *S. aureus* infections (SAI) in Germany were conducted in single centers, had relatively short follow-up periods [15, 16], reported SSI rates regardless of pathogen [1, 17]*,* or focused on specific subgroups [18, 19]. This study sought to further understand the incidence, risk factors and clinical and economic outcomes of SAI following orthopedic surgeries using a large German claims dataset.

## Methods

### Study design and population

A retrospective, non-interventional cohort analysis was conducted based on anonymized claims data provided by AOK PLUS, a German statutory health insurance fund with approximately 3.2 million insured people in the German federal states of Saxony and Thuringia. This work builds upon a previous abstract presented at The European Bone and Joint Infection Society (EBJIS) Meeting [20]. The analysis included patients who were continuously insured by the sickness fund from 2011 to 2016. A patient’s index date was defined as the first inpatient endoprosthetic hip or knee surgery, or spine surgery (German operational procedure codes (OPS): 5–820, 5–821, 5–822, 5–823, 5–83), between January 1, 2012 and December 31, 2015. Patients were excluded based on the following criteria: (i) age < 18 years at index date; (ii) any other surgery in the 180 days before index date; (iii) any surgery performed on the same part of the body (knee/hip/spine) as the index surgery in the 365 days baseline period before index date; (iv) any SAI documented in the 90 days before index date; and (v) more than one type of surgery of interest (knee/hip/spine) during the index hospitalization. Inpatient and outpatient ICD-10 diagnosis codes were used to identify SAI up to 365 days after the index surgery: A41.0 (sepsis due to *S. aureus*); U80.0 (*S. aureus* with resistance to antibiotics); B95.6 (*S. aureus* as cause of a disease that is classified elsewhere).

Incidence was assessed during index hospitalization, 30, 90, 180 and 365 days after the index surgery and separately by index surgery type. Cumulative incidence was calculated as percentage of patients with SAI. Incidence density was calculated as the number of infected patients per 1000 person-years (PY). Only the first SAI identified after the index surgery was counted towards incidence. Kaplan-Meier curves were used to assess the time without SAI, censoring for death. A sensitivity analysis was conducted additionally censoring for any follow-up surgery at a different location of the body from the index surgery.

For risk factor and outcomes analyses, patients with at least one post-surgical SAI were compared with patients without post-surgical SAI but who may have had infections caused by other pathogens during the 365 days following the index surgery. Risk factors of post-surgical SAI were assessed using multivariable Cox regression analysis with time until first observed SAI as the dependent variable. Independent variables included were identified a priori: age at the index surgery; gender; index surgery type (hip, knee, spine); length of index hospitalization in days; complications due to orthopedic prosthetic devices/implants during index hospitalization; bacterial infections (not caused by any *Staphylococci*) during index hospitalization, Charlson Comorbidity Index (CCI) during 365-days pre-index period [21]; number of antibiotic prescriptions (pre-index period); and previous fractures at the same body part as the index surgery (pre-index period).

All-cause mortality, number of general practitioner visits or specialist visits per PY, number of follow-up hospitalizations and length of hospital stays, prescribed defined daily doses (DDD) of outpatient antibiotic agents (as defined by World Health Organization) [22], and direct healthcare costs were assessed up to 365 days after the index surgery. All-cause direct healthcare costs were calculated based on diagnosis-related group reimbursements for inpatient hospitalizations, official retail list prices for outpatient medication prescriptions, and documented ‘treatment points’ for outpatient physician visits [23]. For the outpatient diagnoses, only the quarter of the year was available and therefore date of diagnosis was assumed to be middle of the applicable quarter.

### Statistical analysis

Differences in baseline characteristics between patients who experienced or did not experience a SAI were calculated using either Pearson chi-squared test, Mann-Whitney U test, t-test and Kruskal – Wallis test. Unadjusted incidence rates (IR) and incidence rate ratios (IRR) were calculated for the comparison of outcomes between SAI and non-SAI groups. Multivariable Cox regression analyses were used to assess the hazard ratios (HRs) of death and hospitalizations, respectively. A multivariable generalized linear model (GLM, gamma distribution, log link function) was used to assess whether SAI were associated with greater cost compared with the non-SAI group. Potential cofounders within all multivariable models were defined a priori and excluded if they did not reach the significance level of *p* < 0.10 based on stepwise backward elimination. All reported *p*-values were two-sided and 95% confidence intervals (CI) were calculated for HRs. Descriptive evaluations were carried out with Microsoft SQL Server 2008 and Microsoft Excel 2010. All other statistical analyses were carried out using Stata version 14.1 software (StataCorp. 2015. *Stata Statistical Software: Release 14*. College Station, TX: StataCorp LP).

## Results

**Fig. 1:**
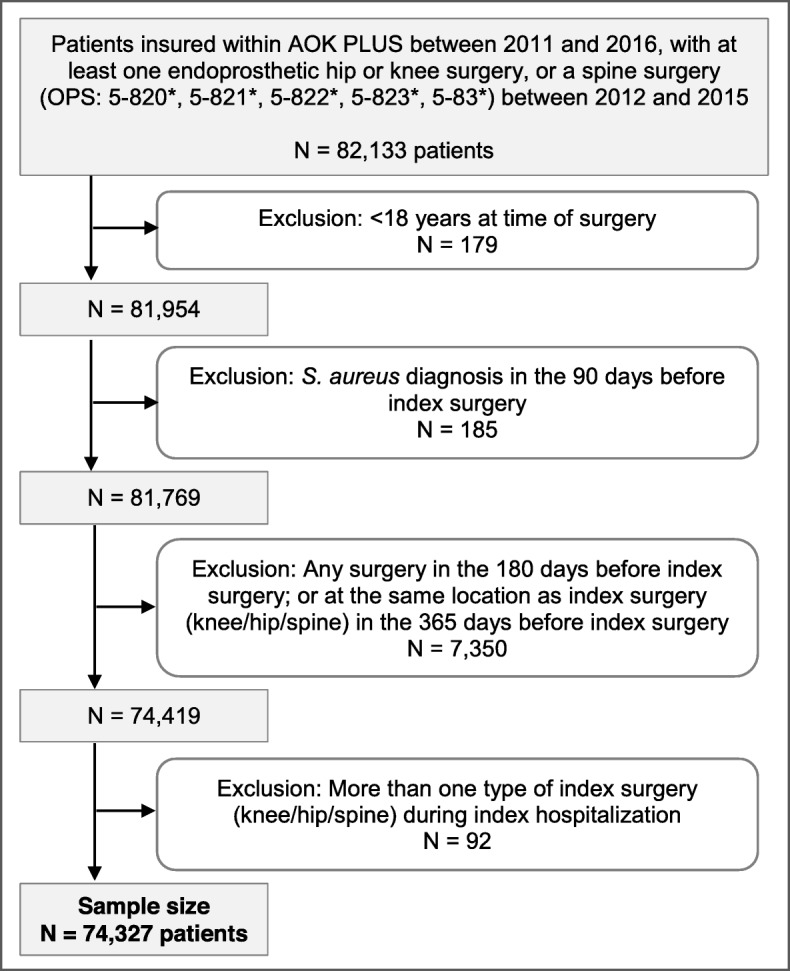
Patient attrition chart presenting numbers of included and, afterwards, stepwise excluded patients based on the defined exclusion criteria. *S. aureus, Staphylococcus aureus*

Overall, 74,327 patients met all defined inclusion criteria (Fig. 1). The mean age at time of surgery (index date) was 69.59 years (standard deviation [SD]: 13.41 years); 61.58% were female and the mean CCI was 2.28 (SD: 2.42). Of all patients, 29,429 (39.6%) underwent an endoprosthetic hip; 21,285 (28.6%) an endoprosthetic knee; and 23,613 (31.8%) a spine surgery. According to ICD-10 codes, the majority of knee and hip surgeries were primary surgeries rather than revision/exchange or removal surgeries; 72.24% of the spine surgeries were spinal fusion procedures. On average, the length of stay for index hospitalization was 14.07 days (SD: 9.12); 5.78% of patients experienced complications of prosthetic devices and implants during index hospitalization and 5.50% had bacterial infections caused by pathogens other than Staphylococci during their index hospitalization. In total, 21.69% experienced fractures at the location of the respective index surgery in the 3 months before the index surgery (Table 1).

**Table 1:** Baseline characteristics of observed patients

***Characteristic***	***All observed patients***	***Patients with a hip surgery***	***Patients with a knee surgery***	***Patients with a spine surgery***	***Patients with SAI in the 365-d follow-up***	***Patients w/o SAI in the 365-d follow-up***	***p-value for differences between patients with/without SAI***
N	74,327	29,429	21,285	23,613	1430	72,897	
Age in years; Mean (SD)	69.59 (13.41)	74.09 (11.63)	70.39 (9.56)	63.25 (15.79)	74.97 (11.54)	69.48 (18.42)	*p* < 0.001
Female sex; N (%)	45,771 (61.58)	18,912 (64.26)	13,915 (65.37)	12,944 (54.82)	781 (54.62)	44,990 (61.72)	*p* < 0.001
CCI (12-month baseline period); Mean (SD)	2.28 (2.42)	2.48 (2.48)	2.15 (2.15)	2.14 (2.56)	3.76 (2.95)	2.25 (2.40)	*p* < 0.001
Length of index hospitalization in days; Mean (SD)	14.07 (9.12)	15.37 (9.08)	13.12 (6.04)	13.29 (11.09)	20.05 (15.27)	13.95 (8.92)	*p* < 0.001
Type of index surgery							
Primary surgery; N (%)	45,706 (61.49)	26,427 (89.80)	19,279 (90.58)	–	816 (57.06)	44,890 (61.58)	*p* < 0.001
Revisional/exchange/removal surgery; N (%)	3874 (5.21)	2265 (7.70)	1609 (7.56)	–	116 (8.11)	3758 (5.12)	*p* < 0.001
Unspecified^a^; N (%)	1134 (1.53)	737 (2.50)	397 (1.87)	–	36 (2.52)	1098 (1.51)	*p* = 0.002
Spinal fusion; N(%)	6554 (8.82)	–	–	6554 (27.76)	139 (9.72)	6415 (8.80)	*p* = 0.224
Other spine surgeries; N(%)	17,059 (22.95)	–	–	17,059 (72.24)	323 (22.59)	16,736 (22.96)	*p* = 0.741
Complications due to prosthetic devices/ implants/grafts during index hospitalization; N (%)	4298 (5.78)	2405 (8.17)	1730 (8.13)	163 (0.69)	131 (9.16)	4167 (5.72)	*p* < 0.001
Recent fractures at the location of index surgery (3 months baseline period); N (%)	16,123 (21.69)	8963 (30.46)	592 (2.78)	6568 (27.82)	627 (43.85)	15,496 (21.26)	*p* < 0.001
Bacterial infections (not caused by any Staphylococci) during index hospitalization; N (%)	4091 (5.50)	2142 (7.28)	644 (3.03)	1305 (5.53)	208 (14.55)	3883 (5.33)	*p* < 0.001
Outpatient prescriptions of antibiotics (12 months baseline period); Mean (SD)	0.60 (1.18)	0.56 (1.17)	0.61 (1.14)	0.64 (1.24)	0.84 (1.57)	0.60 (1.17)	*p* < 0.001
Follow-up period within 365 days follow-up in days, censored at; Mean (SD)							
Death	351.34 (61.32)	341.98 (79.29)	362.33 (26.45)	353.09 (56.11)	324.46 (88.71)	351.86 (60.54)	*p* < 0.001
Death or infection date	347.95 (67.65)	337.93 (84.89)	359.81 (36.66)	349.74 (62.93)	148.23 (101.97)	351.86 (60.54)	*p* < 0.001
Death, infection date or date of any next surgery not performed at the same body location as index surgery	318.28 (100.74)	313.66 (107.17)	335.05 (80.22)	308.93 (107.07)	120.93 (94.73)	322.15 (96.92)	*p* < 0.001

*CCI* Charlson Comorbidity Index, *S. aureus, Staphylococcus aureus*;*, SAI Staphylococcus aureus* infection, *SD* Standard deviation, *w/o* without

^a^Knee and hip surgeries for which both, implementation and revision/exchange/removel OPS-codes were coded

**Fig. 2:**
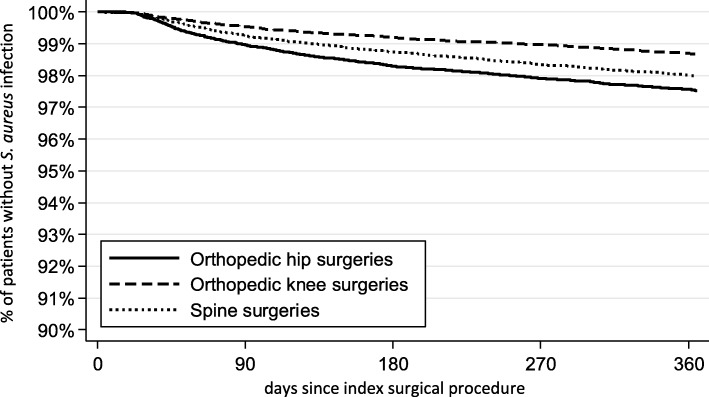
Kaplan-Meier curves for the percentage of patients without *S. aureus* infections (or SSIs) after an orthopaedic surgery. Differences between the surgery groups was analysed using log rank test (*p* < 0.001). *S. aureus, Staphylococcus aureus*

Overall, 1.92% of observed patients (20.20 SAI per 1000 PY) experienced a SAI in the 365-day follow-up period. The percentages of patients experiencing a SAI within 30, 90 and 180 days after the index surgery date were 0.13, 0.77, and 1.26%, respectively (Fig. 2). Incidence varied by surgery type with the highest 365-day incidence among hip surgery patients (2.33%; 25.23 infections per 1000 PY), followed by spine surgery patients (1.74%; 20.43 infections per 1000 PY) and knee surgery patients (1.32%; 13.40 infections per 1000 PY) (Table 2). Incidence of SAI was higher among surgery patients with recent fractures than in those without (Supplementary Table 2). Overall, mean time until infection was 148.23 days (95% CI: 142.94–153.52); median time was 120.50 days. Results of bivariable analyses of patient characteristics between groups are presented in Table 1.

**Table 2:** Incidence of *S. aureus* infections

***N***	***All endoprosthetic surgeries***	***Hip surgeries***	***Knee surgeries***	***Spine surgeries***
	***74,327***	***29,429***	***21,285***	***23,613***
Within index hospitalization				
N (%)	7 (0.01)	2 (0.01)	1 (0.00)	4 (0.02)
per 1000 py	2.63	1.73	1.42	5.04
Within 30 days				
N (%)	93 (0.13)	50 (0.17)	17 (0.08)	26 (0.10)
per 1000 py	15.35	21.01	9.73	13.46
Within 90 days				
N (%)	570 (0.77)	296 (1.01)	98 (0.46)	176 (0.66)
per 1000 py	31.76	42.31	18.77	30.71
Within 180 days				
N (%)	940 (1.26)	481 (1.63)	169 (0.79)	290 (1.09)
per 1000 py	26.50	35.02	16.25	25.60
Within 365 days				
N (%)	1430 (1.92)	687 (2.33)	281 (1.32)	462 (1.74)
per 1000 py	20.20	25.23	13.40	20.43

*py* Patient years, S *aureus, Staphylococcus aureus*

In the sensitivity analysis censoring for death and follow-up surgery performed at a different location of the body than the index surgery, the calculated incidence among these patients within 365 days of the index surgery was 9.23 cases per 1000 PY (0.8%). Within 30, 90 and 180 days of the index surgery the IR was 8.13 (0.07%), 15.38 (0.36%), and 12.72 (0.58%) per 1000 PY, respectively (Supplementary Table 1).

Risk factors for post-surgical SAI which retained significance (*p* < 0.05) in the multivariable Cox regression analysis were: recent fractures at the location of the index surgery (HR = 2.05), other bacterial infections during index hospitalizations (HR = 1.38), complications with devices/implants during index hospitalization (HR = 1.27), higher CCI (HR = 1.13), number of previous antibiotic prescriptions (HR = 1.06), older age (HR = 1.02) and longer index hospitalization stay (HR = 1.02) (Table 3). Knee surgeries (HR = 0.84) were associated with a lower risk compared with hip or spine surgeries; as was female sex (HR = 0.62).

**Table 3:** Risk Factors associated with *S. aureus* infection: Multivariable Cox regression analysis results

***Variable***	***Patients who experienced a SAI in the 365-day follow-up period (N = 1430) compared to patients who did not experience a SAI (N = 72,897)***		
	***HR***	***95% CI***	***p-value***
Age at index	1.02	1.01–1.02	*p* < 0.001
Female sex	0.62	0.56–0.70	*p* < 0.001
Index surgery type			
Hip; N (%)	- Reference -		
Knee; N (%)	0.84	0.73–0.98	*p* = 0.024
Spine; N (%)	0.97	0.86–1.10	*p* = 0.686
CCI (12 months baseline period)	1.13	1.11–1.15	*p* < 0.001
Length of index hospitalization in days	1.02	1.01–1.02	*p* < 0.001
Complications due to prosthetic devices/ implants/grafts during index hospitalization	1.27	1.05–1.54	*p* = 0.012
Recent fractures at the location of index surgery (3 months baseline period)	2.05	1.81–2.32	*p* < 0.001
Bacterial infections (not caused by any Staphylococci) during index hospitalization	1.38	1.16–1.64	*p* < 0.001
Number of outpatient prescriptions of antibiotics (12 months baseline period)	1.06	1.03–1.09	*p* < 0.001

HRs are based on a conducted multivariable Cox regression analysis with time to first *S. aureus* infection after index surgery as the dependent variable. *CCI* Charlson Comorbidity Index, S *aureus, Staphylococcus aureus, SAI Staphylococcus aureus* infection

**Fig. 3:**
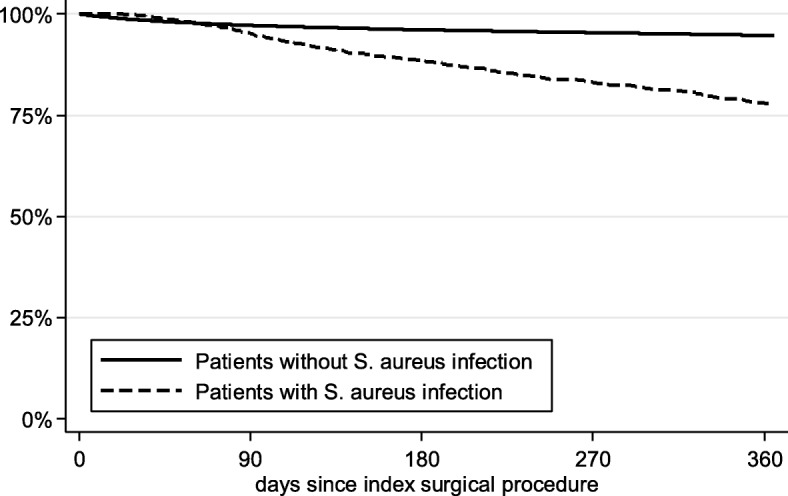
Kaplan-Meier curves for the percentage of patients alive after an orthopaedic surgery. Differences between the groups was analyzed using log rank test (*p* < 0.001). S. aureus, *Staphylococcus aureus*

**Fig. 4:**
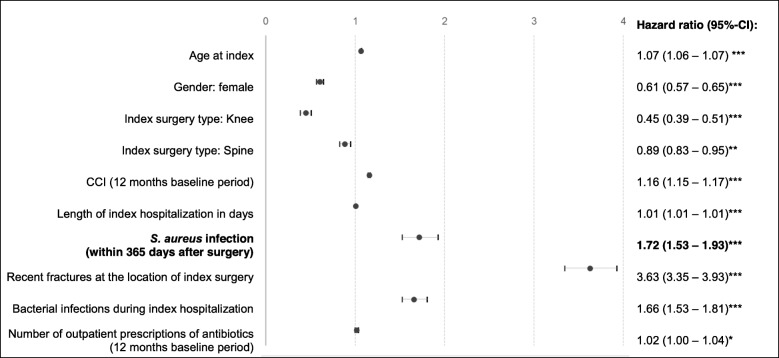
Multivariable Cox regression analysis results for time until death. Number of subjects = 74,327; Number of events = 4191; LR chi^2^ = 9113.15 (*p* < 0.001). ***p < 0.001; **p < 0.10; *p < 0.050

Table 4 shows all-cause mortality, healthcare resource utilization and all-cause direct healthcare costs for SAI versus non-SAI surgery patients. The proportion of patients who died within 90 days of surgery was 4.90% versus 2.79% among SAI versus non-SAI groups, respectively (*p* < 0.001). This pattern persisted for mortality within 180 days (11.61% vs. 3.91%; *p* < 0.001) and 365 days post-surgery (22.38% vs. 5.31%, *p* < 0.001). Over 365 days, the mortality risk was 1.72 times higher in the SAI group than the non-SAI group when adjusting for confounders (95% CI: 1.53–1.93; *p* < 0.001) (Fig. 3). Further factors that were significantly associated (*p* < 0.05) with increased risk of death were male sex (HR = 0.61 for females), older age (HR = 1.07), higher CCI (HR = 1.16), longer index hospitalization (HR = 1.01), recent fractures (HR = 3.63), other bacterial infection (HR = 1.66) and previous number of antibiotic prescriptions (HR = 1.02). Knee (HR = 0.45) and spine surgeries (HR = 0.89) were associated with a lower mortality risk, when compared with hip surgeries (Fig. 4).

**Table 4:** All-cause mortality, HCRU and costs after orthopedic surgery in patients with/without *S. aureus* infection

	***Patients with SAI in the 365-d follow-up***	***Patients w/o SAI in the 365-d follow-up***	***p-value for differences between patients with/without SAI***
N	1430	72,897	
**Patients who died**			
During index hospital stay,%	0	1.27	*p* < 0.001
Within 30 d, %	0.35	1.40	*p* < 0.001
Within 90 d, %	4.90	2.79	*p* < 0.001
Within 180 d, %	11.61	3.91	*p* < 0.001
Within 365 d, %	22.38	5.31	*p* < 0.001
**365-d follow-up HCRU per patient-year**			
No. of outpatient GP visits, Mean (SD)	3.17	3.20	*p* = 0.566
No. of outpatient specialist visits, Mean (SD)	3.65	3.46	*p* < 0.001
No. of all-cause hospitalizations, Mean (SD) ^a^	3.06	0.70	*p* < 0.001
No. of hospital days, Mean (SD) ^a^	53.48	6.92	*p* < 0.001
No. of outpatient prescriptions of antibiotics, Mean (SD)	2.17	0.63	*p* < 0.001
**365-d follow-up costs (€) per patient-year**			
Outpatient physician visits	1149€	1011€	*p* < 0.001
Outpatient all-agent medication	2605€	1327€	*p* < 0.001
Hospitalizations (incl. Inpatient drug treatment) ^b^	39,080€	11,444€	*p* < 0.001
Total costs	42,834€	13,781€	*p* < 0.001

Outcomes measured within 365 days after index surgical procedure, censoring at time of death

*S aureus, Staphylococcus aureus, SAI Staphylococcus aureus* infection, *SD Standard deviation*

^a^Excluding index hospitalization; ^b^Including index hospitalization

Healthcare resource utilization and costs were significantly higher for SAI patients versus non-SAI patients (Table 4). The number of hospitalization days per patient-year in the 365-day follow-up period was 7.73 times higher for SAI patients versus non-SAI patients when excluding the index surgery hospitalization (53.48 days vs. 6.92 days; *p* < 0.001). Adjusting for confounders, the risk of first re-hospitalization was 4.21 times greater among SAI patients than non-SAI patients. Mean overall direct healthcare costs per PY were 42,834€ for the SAI group versus 13,781€ for the non-SAI group (cost ratio: 3.11, *p* < 0.001) (Table 4). Inpatient hospitalization costs accounted for 91 and 83% of the overall costs among the SAI group and non-SAI group, respectively. After adjusting for confounders, SAI patients experienced 1.93 times the direct healthcare costs within 365 days of the index surgery compared with non-SAI patients (*p* < 0.001) (Table 5).

**Table 5:** Generalized linear model of total costs within 365 days follow-up period

***Variable***	***Coef.***	***exp(b)***	***p-value***	***95% CI*** ***of exp (b)***
Age at index	0.01	1.01	< 0.001	1.00–1.01
Sex				
*Male*	Reference			
*Female*	−0.16	0.85	< 0.001	0.82–0.87
Index surgery type				
*Hip*	Reference			
*Knee*	−0.06	0.94	0.002	0.90–0.97
*Spine*	0.03	1.04	0.066	0.99–1.07
CCI (12 months baseline period)	0.09	1.10	< 0.001	1.09–1.10
Length of index hospitalization in days	0.01	1.01	< 0.001	1.00–1.00
Complications due to prosthetic devices/ implants/grafts during index hospitalization	0.19	1.21	< 0.001	1.14–1.29
***S. aureus*infection within 365 days after surgery**	**0.66**	**1.93**	**< 0.001**	**1.73–2.13**
Recent fractures at the location of index surgery (3 months baseline period)	0.78	2.19	< 0.001	2.10–2.27
Bacterial infections (not caused by any Staphylococci) during index hospitalization	0.67	1.96	< 0.001	1.83–2.10
Number of outpatient prescriptions of antibiotics (12 months baseline period)	0.03	1.03	< 0.001	1.01–1.03

Number of subjects = 74,327

*CCI* Charlson Comorbidity Index, *CI* Confidence interval, *Coef* Coefficient, *S aureus, Staphylococcus aureus*

## Discussion

This study assessed the burden of post-surgical SAI among orthopedic surgery patients in Germany. We identified a similar near term SAI infection risk as a prior study in Germany [5]. In our analysis 0.77% of patients were infected by *S. aureus* within 90 days of index endoprosthetic surgery (0.46% for knee surgeries; 1.01% for hip surgeries; 0.66% spine surgeries). In previous German studies, 0.98% of patients who underwent orthopedic surgeries acquired SSI, with approximately one-third of SSI caused by *S. aureus* [5, 9]. As late SSIs (e.g. 180 days, 365-days) were not assessed in these German studies, we cannot directly compare our SSI results [24]. However, our 90-day post-surgery SAI incidence of 0.77% is similar to results from a United States study assessing post elective orthopedic surgery SAI of 0.8% [6]. Our study assessed a longer follow-up period after the index surgery (up to 365 days) in order to capture late deep infections, which has not previously been assessed. To ascertain whether SAIs are related to surgeries other than the index orthopedic surgery, we performed a sensitivity analysis censoring at time of a follow-up surgery not conducted on the same body part and found SAI incidence reduced by 50%. We did not censor for surgeries that were done on the same body part, given that some revision surgeries could have occurred due to a SAI. We recognize using retrospective database did not allow to further determine whether the SAI came from the index surgery or as a result of a follow-up surgery. Therefore, we did not censor for any follow-up surgeries in the risk factor or outcomes analyses. When evaluating the impact of infection risk reduction or prevention methods that could have implications across multiple surgeries within a given time period (e.g. smoking cessation and vaccines) understanding the SAI impact regardless of causative surgery is helpful to assess the potential overall impact of the infection prevention intervention. To further inform this, we assessed the incidence of SAI among those with recent fractures at the location of the index surgery and those without, assuming recent fractures to be most indicative of urgent or emergent surgeries and therefore less likely to allow for implementation of infection prevention activities that require advance time (e.g., smoking cessation, vaccinations).

We identified risk factors for SAI consistent with those reported in the literature for orthopedic SSIs [25]. Older patients and male patients were found to have a higher risk for infection after orthopedic surgery, consistent with previous literature [2, 25, 26]. In addition, the IR of SAI was higher in patients who underwent a hip surgery compared with those patients undergoing a knee surgery, in line with previous studies [2, 26]. Lai et el. found that the presence of more than 2 comorbidities can increase the risk of SSI [27]; our study further supports this by reporting that a higher number of comorbidities (measured as CCI) had a significant impact and increases the SAI risk. Other bacterial infections and longer index hospitalization stay were also found to increase the risk for infections, both in our study and in existing literature [25]. We identified some additional risk factors such as complications with devices/implants during index hospitalization, recent fractures at the location of the index surgery, and number of previous antibiotic prescriptions. Nevertheless, we did not assess the SAI risk associated with other known risk factors, such as obesity, body mass index multiple follow-up hospitalizations or additional surgeries.

In our study, the length of index hospitalization was higher in patients with SAI compared with those without SAI (20.05 vs. 13.95 days). Also, hospitalization days during 365-days follow-up period were substantially higher (76.04 vs. 21.39 days). This is in line with previous observations in Germany, reporting considerable differences in the length of stay between infected and non-infected patients [28, 29]. Compared with previous studies in the United States [3, 30, 31], our hospitalization day results are higher. This reflects differences in the health care practice and reimbursement systems of both states which also account for a longer length of stays in the hospitals in Germany compared to the United States [32]. Our results showed overall unadjusted cost ratio within 365 days after index orthopedic surgery of 3.1 among SAI compared with non-SAI patients (*p* < 0.001). A slightly higher cost ratio (3.7) was seen in patients who underwent knee arthroplasty in a German hospital setting and experience/did not experience any SSI [29, 30].

The results for 90-day post-surgery mortality (2.79% in the non-*S. aureus* and 4.90% in the *S. aureus* group) are comparable to previous studies conducted in the United States, reporting death rates of 1.5–3% for non-SAI patients, 6.7–20.7% for SAI-patients during 90-day follow-up after any surgery [6, 33, 34]. Razavi et al. reported death rates of 6.6%/16.8% within 180 days of orthopedic surgery, which is slightly higher than the reported mortality of 3.9%/11.6% in our study [2]. However, our reported mortality risk ratio within 180 days after any orthopedic surgery (3.05) is slightly higher than that publication (2.56) [2].

This study has some limitations. First, generalizability could be affected by the fact that the health insurance fund only covers patients in two regions of Germany (Saxony and Thuringia). However, since health reimbursement rules are identical across Germany, considerable differences in the treatment of patients are unlikely and therefore results are expected to be generalizable within Germany, but not outside of Germany, by the authors. Given the lack of laboratory data, identification of SAI was limited to documented outpatient and inpatient diagnosis codes (ICD-10), which may have introduced misclassification bias and underestimated the true SAI in the real world. Moreover, underestimation might have happened since patients could have died of SAI after the index hospitalization, without being diagnosed as such.

The index surgery date was not available, therefore the hospital admission date was used as the index date, which could have caused overestimation of the time until infection, and hospitalization days attributable to SAI. Since the specific date when the SAI was identified was not available either, we may have the approximate time to infection in this study, particularly for outpatients who only had data on the quarterly basis on the timing of SAI diagnosis. Although some risk factors for SAI could also be risk factors for dying, death was not included as a covariate in our Cox regressions as a competing risk factor. Ultimately this might have caused overestimation of SAI risk.

Moreover, the correlation between length of hospitalization stay and SAI risk as identified in the analysis might be biased in that SAIs might themselves lead to increased hospitalization time for the index stay, even though they might not have been diagnosed at that time. Finally, when examining the association between index surgery with SAI, any actual association between the index surgery and following SAI is presumed, especially since no specific ICD-10 code classifying an infection as a SSI is available in our dataset. Although an association can be assumed with some degree of certainty for early infections. Infections observed during longer follow-up periods might be attributable to other factors. To account for this, sensitivity analyses were performed for incidence with censoring for follow-up surgeries performed on a different body part from the index surgery. Surgeries on the same part of the body were not censored, as these might have been revision surgeries caused by SAI.

## Conclusions

As a major pathogen causing postsurgical infections following orthopedic surgeries, SAI continues to burden patients and healthcare systems in Germany, despite current infection control measures. Our results suggest that improvements in infection control, risk reduction and prevention methods which further prevent SAIs could improve clinical and economic outcomes after orthopedic surgeries.

## Supplementary information

**Additional file 1: Table S1.** Sensitivity analysis - Incidence of *S. aureus* infections, censored at time of death or any follow-up surgery not performed on location of the index surgery.

**Additional file 2: Table S2.** Incidence of *S. aureus* infections in patients with/without recent fractures.

## Data Availability

The data that support the findings of this study are abstracted from individual patient records. Data were available for research purposes from the sickness fund upon request, in an anonymized form. Due to restrictions around revealing patients’ confidential information, data were used under license for the current study, and so are neither publicly available nor can be shared further.

## Abbreviations

CCI: Charlson Comorbidity Index
CI: Confidence interval
Exp(b): Exponentiated coefficient b
HR: Hazard ratio
HCRU: Healthcare resource utilization
ICD-10: International Classification of Diseases and Related Health Problems
IR: Incident rate
IRR: Incident rate ratio
PY: Patient-year
SD: Standard deviation
*S. aureus*: *Staphylococcus aureus*
SAI: *Staphylococcus aureus* infections
SSI: Surgical site infection
