# Supplementary material for: Burden of Staphylococcus aureus infections after orthopedic surgery in Germany
Source: BMC Infect Dis. 2020 Mar 19;20:233. doi: 10.1186/s12879-020-04953-4 (PMC7082972; doi:10.1186/s12879-020-04953-4)
Supplement: Supplementary file 1 — Additional file 1: Table S1. Sensitivity analysis - Incidence of S. aureus infections, censored at time of death or any follow-up surgery not performed on location of the index surgery. [file 12879_2020_4953_MOESM1_ESM.docx]

**Supplementary Table 1:** Sensitivity analysis - Incidence of S. aureus infections, censored at time of death or any follow-up surgery not performed on location of the index surgery

|  |  | ***All endoprosthetic surgeries*** | ***Hip***  ***surgeries*** | ***Knee surgeries*** | ***Spine surgeries*** |
| --- | --- | --- | --- | --- | --- |
| ***N*** |  | ***74,327*** | ***29,429*** | ***21,285*** | ***23,613*** |
|  |  |  |  |  |  |
| Within index hospitalization |  |  |  |  |  |
| N (%) |  | 7 (0.01) | 2 (0.01) | 1 (0.00) | 4 (0.02) |
| per 1,000 py |  | 2.63 | 1.73 | 1.42 | 5.04 |
|  |  |  |  |  |  |
| Within 30 days |  |  |  |  |  |
| N (%) |  | 49 (0.07) | 31 (0.11) | 10 (0.05) | 8 (0.03) |
| per 1,000 py |  | 8.13 | 13.10 | 5.75 | 4.17 |
|  |  |  |  |  |  |
| Within 90 days |  |  |  |  |  |
| N (%) |  | 270 (0.36) | 160 (0.54) | 39 (0.18) | 71 (0.27) |
| per 1,000 py |  | 15.38 | 23.34 | 7.59 | 12.78 |
|  |  |  |  |  |  |
| Within 180 days |  |  |  |  |  |
| N (%) |  | 431 (0.58) | 247 (0.84) | 65 (0.31) | 119 (0.45) |
| per 1,000 py |  | 12.72 | 18.69 | 6.46 | 11.21 |
|  |  |  |  |  |  |
| Within 365 days |  |  |  |  |  |
| N (%) |  | 598 (0.80) | 340 (1.16) | 92 (0.43) | 166 (0.62) |
| per 1,000 py |  | 9.23 | 13.45 | 4.71 | 8.31 |
|  |  |  |  |  |  |

py = patient years; S. aureus, Staphylococcus aureus.
