# Supplementary material for: Burden of Staphylococcus aureus infections after orthopedic surgery in Germany
Source: BMC Infect Dis. 2020 Mar 19;20:233. doi: 10.1186/s12879-020-04953-4 (PMC7082972; doi:10.1186/s12879-020-04953-4)
Supplement: Supplementary file 2 — Additional file 2: Table S2. Incidence of S. aureus infections in patients with/without recent fractures. [file 12879_2020_4953_MOESM2_ESM.docx]

**Supplementary Table 2:** Incidence of S. aureus infections in patients with/without recent fractures

|  |  | ***All endoprosthetic surgeries*** | | ***Hip surgeries*** | | ***Knee surgeries*** | | ***Spine surgeries*** | |
| --- | --- | --- | --- | --- | --- | --- | --- | --- | --- |
| ***Recent location-related fractures*** |  | ***w fractures*** | ***w/o fractures*** | ***w fractures*** | ***w/o fractures*** | ***w fractures*** | ***w/o fractures*** | ***w fractures*** | ***w/o fractures*** |
| ***N*** |  | ***16,123*** | ***58,204*** | ***8,963*** | ***20,466*** | ***592*** | ***20,693*** | ***6,568*** | ***17,045*** |
|  |  |  |  |  |  |  |  |  |  |
| Within index hospitalization |  |  |  |  |  |  |  |  |  |
| N (%) |  | 4 (0.02) | 3 (0.01) | 2 (0.02) | 0 (0.00) | 0 (0.00) | 1 (0.00) | 2 (0.03) | 2 (0.01) |
| per 1,000 py |  | 5.45 | 1.56 | 4.70 | 0.00 | 0.00 | 1.47 | 7.16 | 3.89 |
|  |  |  |  |  |  |  |  |  |  |
| Within 30 days |  |  |  |  |  |  |  |  |  |
| N (%) |  | 37 (0.23) | 56 (0.10) | 26 (0.29) | 24 (0.12) | 0 (0.00) | 17 (0.08) | 11 (0.17) | 15 (0.09) |
| per 1,000 py |  | 28.80 | 11.73 | 37.03 | 14.31 | 0.00 | 10.01 | 20.59 | 10.74 |
|  |  |  |  |  |  |  |  |  |  |
| Within 90 days |  |  |  |  |  |  |  |  |  |
| N (%) |  | 271 (1.68) | 299 (0.51) | 180 (2.01) | 116 (0.57) | 8 (1.35) | 90 (0.43) | 83 (1.26) | 93 (0.55) |
| per 1,000 py |  | 73.28 | 20.98 | 90.55 | 23.16 | 56.17 | 17.72 | 59.31 | 22.34 |
|  |  |  |  |  |  |  |  |  |  |
| Within 180 days |  |  |  |  |  |  |  |  |  |
| N (%) |  | 436 (2.70) | 504 (0.87) | 284 (3.17) | 197 (0.96) | 13 (2.20) | 156 (0.75) | 139 (2.12) | 151 (0.89) |
| per 1,000 py |  | 61.28 | 17.78 | 75.32 | 19.77 | 46.42 | 15.41 | 45.36 | 18.27 |
|  |  |  |  |  |  |  |  |  |  |
| Within 365 days |  |  |  |  |  |  |  |  |  |
| N (%) |  | 627 (3.89) | 803 (1.38) | 395 (4.41) | 292 (1.43) | 20 (3.38) | 261 (1.26) | 212 (3.23) | 250 (1.47) |
| per 1,000 py |  | 45.61 | 14.07 | 55.07 | 14.56 | 35.90 | 12.79 | 35.23 | 15.07 |
|  |  |  |  |  |  |  |  |  |  |

py = patient years; *S. aureus, Staphylococcus aureus*; w = with; w/o = without.
